# The effect of perioperative pelvic floor muscle exercise on urinary incontinence after radical prostatectomy: a meta-analysis

**DOI:** 10.1590/S1677-5538.IBJU.2023.0053

**Published:** 2023-05-20

**Authors:** Erkang Geng, Sifan Yin, Yulin Yang, Changxing Ke, Kewei Fang, Jianhe Liu, Daoqi Wang

**Affiliations:** 1 Second Affiliated Hospital of Kunming Medical University Department of Urology Kunming China Department of Urology, the Second Affiliated Hospital of Kunming Medical University Kunming, China

**Keywords:** Prostatic Neoplasms, Urinary Incontinence, Meta-Analysis as Topic

## Abstract

**Background:**

Pelvic floor muscle exercise (PFME) is the most common conservative management for urinary incontinence (UI) after radical prostatectomy (RP). We performed this meta-analysis to investigate whether PFME during the entire perioperative period, including before and after RP, can significantly improve the recovery of postoperative UI.

**Methods:**

We systematically reviewed randomized controlled trials (RCT) from PubMed, Medline, web of science, Cochrane library, and clinicalitrials.com prior to October 2022. Efficacy data were pooled and analyzed using Review Manager Version 5.3. Pooled analyses of urinary incontinence rates 1, 3, 6, and 12 months postoperatively were conducted, using odds ratio (OR) and 95% confidence intervals (CIs).

**Results:**

We included a total of 15 RCT studies involving 2178 patients received RP. Postoperative UI could be improved after 1 month, 3 months and 6 months, and the OR were 0.26 (95%CI:0.15-0.46) 0.30 (95%CI: 0.11-0.80) 0.20 (95%CI: 0.07- 0.56) in postoperative PFME group compared to no PFME group. However, there was no significant difference between the two groups in 12 months after surgery, and the OR was 0.85(95%CI: 0.48,1.51). There were similar results in perioperative PFME group compared to no PFME group with the OR of 0.35 (95%CI: 0.12, 0.98) and 0.40 (95%CI: 0.21, 0.75) in 1 and 3 months after surgery. Our results indicated no significant difference between perioperative PFME group and postoperative PFME group. The OR was 0.58 (95%CI: 0.20-1.71) 0.58 (95%CI:0.20-0.71) and 0.66 (95%CI: 0.32-1.38) in 1, 3 and 6 months after surgery.

**Conclusion:**

Application of PFME after RP significantly reduced the incidence of early postoperative UI, and additional preoperative PFME had no significant improvement on the recovery of UI.

## INTRODUCTION

Prostate cancer is the second most common cancer and the fifth leading cause of cancer death among men, with about 1.4 million new cases and about 375,000 deaths worldwide in 2020 (1). More and more male patients have the opportunity to undergo radical prostatectomy (RP) when prostate cancer is still early and localized with increasing health awareness (2, 3). However, RP can cause some troublesome complications affecting patients’ quality of life, including persistent and troubling urinary incontinence (UI). It was reported that more than 80% of patients suffered from UI one month after RP, and 3-12% still existed UI one year after RP (4). UI after RP is mainly caused by the lack of strength of urethral sphincter (5, 6). Pelvic floor muscle exercise (PFME) (7, 8) is the most common conservative treatment for postoperative UI via continuously contracting and relaxing the pelvic floor muscles to increase pelvic floor muscle tone and improve urinary tract resistance. Previous studies have demonstrated the safety and validity of PFME on UI after RP (6), however, there were a few controversies of when to conduct PFME in recent studies including several reviews (9, 10). We thereby performed this meta-analysis to investigate whether PFME during the entire perioperative period, including before and after RP, can significantly improve the recovery of postoperative UI.

## MATERIALS AND METHODS

### Database searching and literature screening.

A comprehensive literature search was conducted on PubMed, Medline, Web of Science, Cochrane Library and ClinicalTrials.gov, to obtain all relevant English articles published before Oct.2022. The search strategy was: (radical prostatectomy) AND (urinary incontinence) AND (pelvic floor muscle exercise) according to the PICOS principle. Three reviewers screened all the titles and abstracts independently. The language was restricted to English, and articles studying the impact of PFME on recovery of UI after RP were included for further screening. We conducted this meta-analysis according to PRISMA (Preferred Reporting Items for Systematic Reviews and Meta-Analyses).

### Inclusion criteria and exclusion criteria

Articles that met the following criteria were included: (1) Randomized controlled trials (RCTs). (2) Patients were diagnosed with prostate cancer and underwent RP. (3) Patients received pre- or post- operative PFME or no PFME. (4) Studies should report at least one of relevant clinical outcomes of interest (described in data extraction part).

Exclusion criteria: (1) Studies were not in English. (2) Conference abstracts. (3) The interesting data could not be extracted or calculated.

### Data extraction

Data extraction was performed independently by three researchers. We extracted the study’s primary characteristics, including the first author, sample size, year of publication, PFME regimens, follow-up time and incidence of UI. Any differences have been re-solved by discussion.

### Quality evaluation

**Supplementary Figure 2:**
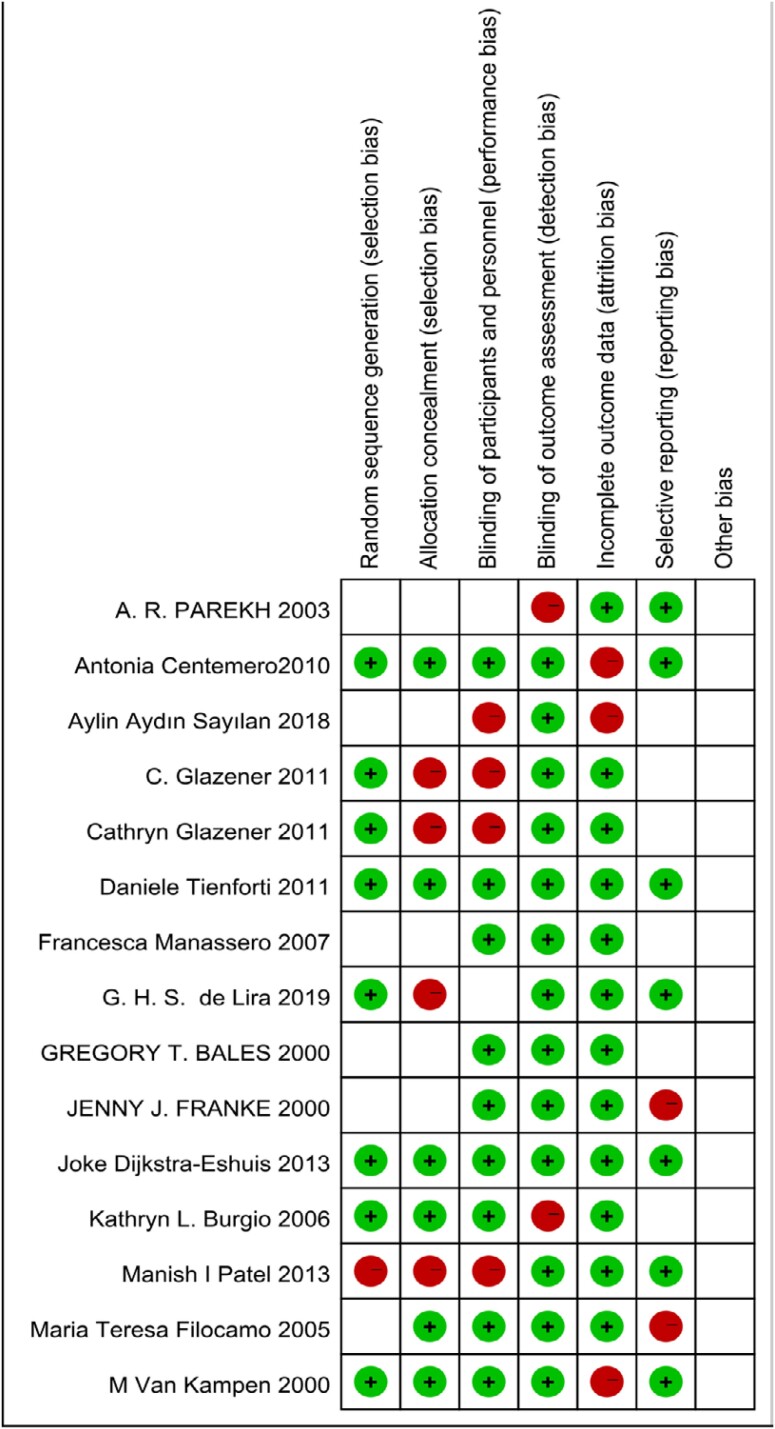
Risk of bias of included studies.

Quality of included studies was evaluated by the Cochrane Collaboration’s tool for assessing risk of bias. The tool consists of seven parts: random sequence generation, allocation concealment, blinding of participants and personnel, blinding of outcome assessment, incomplete outcome data, selective reporting, and other bias. Each part can be graded as low risk of bias, unclear risk of bias, and high risk of bias (Supplementary Figure-2).

Two reviewers carried out this assessment procedure independently and reached a consensus through discussion if disagreements appeared.

### Statistical Analysis

Outcome data were pooled and analyzed with the Review Manager (RevMan) Version 5.3. and presented as odds ratio (OR) with 95% confidence interval (CI) at different follow-up time points. Heterogeneity among studies was evaluated by the I2 test, with I2 > 50% considered to be of significant heterogeneity. In case of significant heterogeneity, random effects model was selected to analyze the outcome data and sensitivity analysis was performed to detect the source of heterogeneity. Intergroup difference was considered to be statistically significant when P < 0.05.

## RESULTS

### Search results

**Figure 1:**
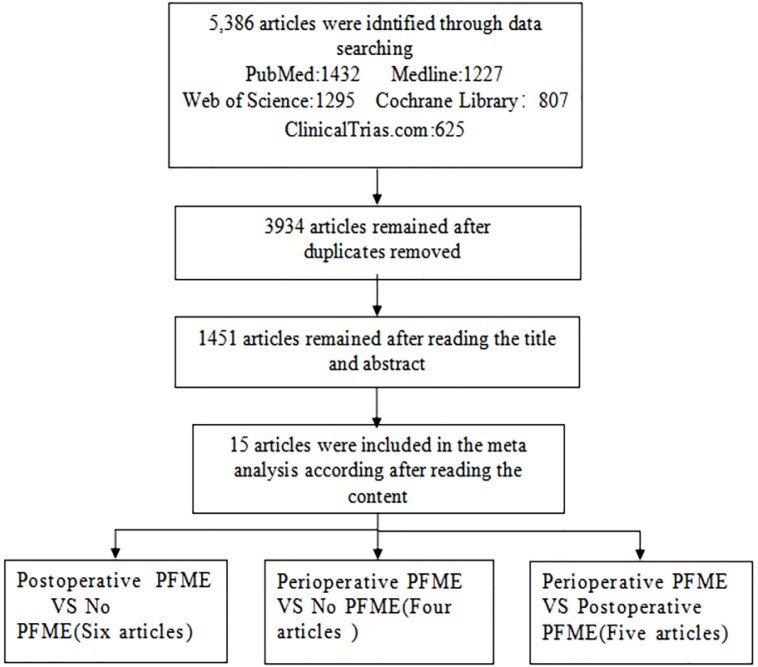
Flow diagram of literature searches according to the preferred reporting items for systematic reviews and meta-analyses statement.

We included a total of 15 RCT studies (11–25), involving 2178 patients (Figure-1). All the articles measured and compared the incidence of UI in different groups, and the follow-up time ranged from 1 month to 1 year. Based on the onset and duration of the PFME, we divided the included studies into three groups: perioperative PFME group postoperative PFME group and no PFME group. Among the 15 studies, 4 studies compared perioperative PFME vs no PFME (11–14), 5 studies compared perioperative PFME vs postoperative PFME (13, 15–18), and 6 compared postoperative PFME vs no PFME (19–24) (Table-1). All included studies used urine pad test to diagnose UI but did not record the degree of urinary incontinence.

**Table 1:** Characteristics of included studies.

Number	First author	Year	Sample size (n)	Type of Research	Experimental group	Control group	Follow-uptime after surgery (month)
1	Burgio et al. (11)	2000	102	RCT	Perioperative PFME	No PFME	1, 3
2	Centemero et al. (15)	2010	118	RCT	Perioperative PFME	Postoperative PFME	1, 3
3	Patel et al. (13)	2013	284	RCT	Perioperative PFME	Postoperative PFME	1, 3
4	Sayılan et al. (14)	2018	60	RCT	Perioperative PFME	No PFME	1, 3, 6
5	Parekh et al. (18)	2003	38	RCT	Perioperative PFME	No PFME	1, 3
6	Glazener et al. (20)	2011	391	RCT	Postoperative PFME	No PFME	12
7	Glazener et al. (22)	2011	397	RCT	Postoperative PFME	No PFME	12
8	Filocamo et al. (19)	2005	300	RCT	Postoperative PFME	No PFME	1, 3, 6
9	Manassero et al. (21)	2007	94	RCT	Postoperative PFME	No PFME	1, 3, 6, 12
10	Dijkstra-Eshuis et al. (16)	2013	102	RCT	Perioperative PFME	Postoperative PFME	6
11	Bales et al. (17)	2000	97	RCT	Perioperative PFME	Postoperative PFME	1, 3, 6
12	Van Kampen et al. (23)	2000	102	RCT	Postoperative PFME	No PFME	1, 3, 6, 12
13	Franke et al. (24)	2000	30	RCT	Postoperative PFME	No PFME	3, 6
14	Tienforti (34)	2011	32	RCT	Perioperative PFME	Postoperative PFME	1, 3, 6
15	de Lira et al. (12)	2019	31	RCT	Perioperative PFME	No PFME	3

### Quality of included studies

**Figure 2:**
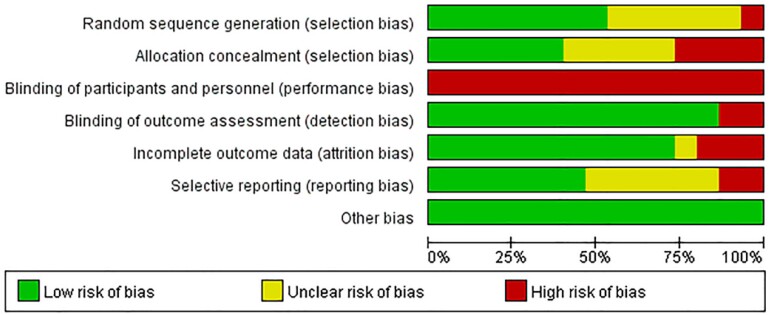
Risk of bias of included studies.

Studies were assessed as moderate-to-high quality according to the Cochrane Collaboration’s tool for assessing risk of bias (Figure-2). Eight studies clarified the method of randomization, and it was reasonable and low risk. Six studies only mentioned randomization without explaining the method, and only one article was high-risk (13). In addition, nearly half of the studies hid the allocation strategy. Studies had a high risk of performance bias caused by the impossibility of participants and personnel blinding. Therefore, we did not exclude studies just because of the high risk of performance bias. In most trials, the data analyst did not know the grouping and treatment of the trial. The follow-up information was detailed described in most studies. There was also no significant publication bias and other bias in these studies.

### Effect of PFME on UI after RP

**Figure 3:**
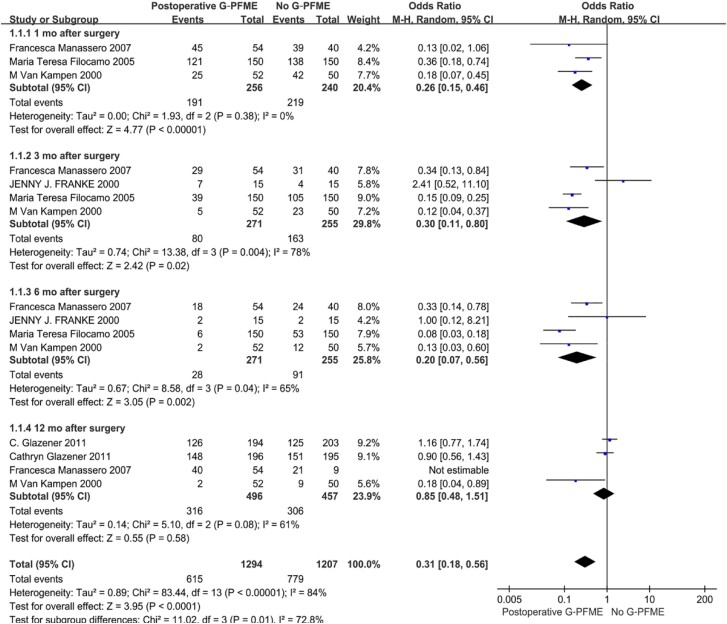
Meta-analysis of objective cure of UI between PFME and No PFME.

**Figure 4:**
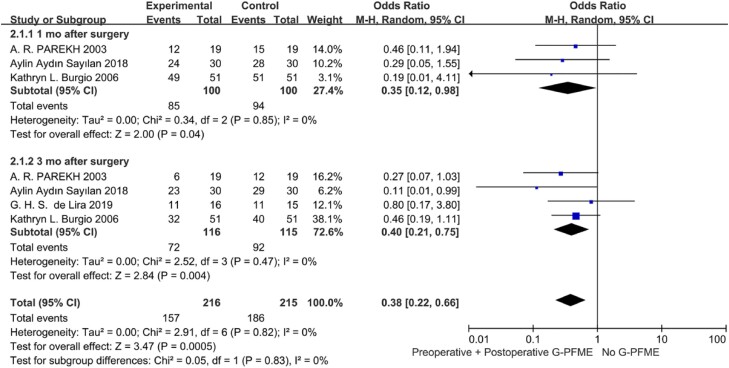
Meta-analysis of objective cure of UI between Perioperative PFME and No PFME.

**Figure 5:**
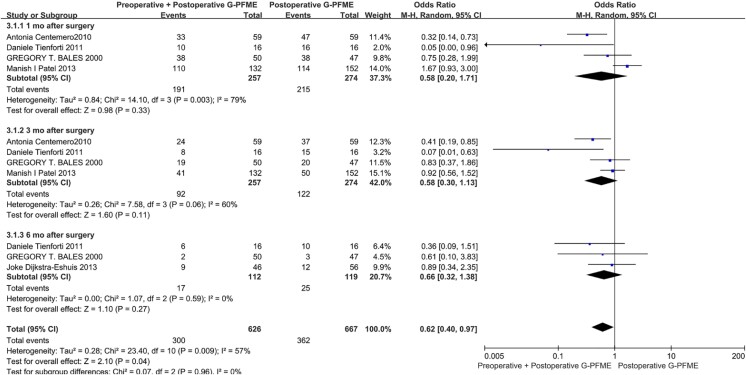
Meta-analysis of objective cure of UI between Perioperative PFME and Postoperative PFME.

Perioperative and postoperative PFME can improve the recovery of UI after RP. The follow-up time points were focused on 1 month, 3 months, 6 months, and 12 months after surgery in our meta-analysis. In the comparison of postoperative PFME with no PFME, postoperative PFME can significantly improve the recovery of postoperative UI in the early and middle postoperative period. Postoperative UI could be relieved after 1 month, 3 months and 6 months, and the OR were 0.26 (95%CI:0.15-0.46, p<0.00001) 0.30 (95%CI: 0.11-0.80, P=0.004) 0.20 (95%CI: 0.07- 0.56, P=0.002). However, there was no significant difference between the two groups after 12 months of surgery, and the OR was 0.8 (95%CI: 0.48,1.51, P=0.58) (Figure-3). We also conducted a comparison between perioperative PFME and no PFME, and the results showed that perioperative PFME could reduce the occurrence of early UI after RP. The OR were 0.35 (95%CI: 0.12, 0.98, P=0.04) and 0.40 (95%CI: 0.21, 0.75, P=0.004), in 1 and 3 months after surgery respectively (Figure-4). We also investigated the perioperative PFME and postoperative PFME, and the results showed that there was no significant statistical difference between these two groups. The OR was 0.58 (95%CI: 0.20-1.71, P=0.33) 0.58 (95%CI:0.20-0.71, P=0.11) and 0.66 (95%CI: 0.32-1.38, P=0.27) at 1, 3 and 6 months after surgery, respectively (Figure-5).

## DISCUSSION

Persistent and disturbing UI is a common complication after RP, with an incidence of 30-40% in early follow-up after surgery. Detrusor overactivity and intrinsic sphincter insufficiency due to sphincteric injury are the most important causes of persistent incontinence after radical prostatectomy. Some reports mention that detrusor overactivity is a significant cause of post-prostatectomy incontinence, others strongly argue that even if other factors play a role, the main cause of UI after RP is intrinsic sphincter deficiency (25, 26). The absence of both functions can be corrected by PFME, which is not only convenient and practical, but also has no other side effects. PFME can increase the activation of tension muscles, then transfer to the functional tasks of other muscles. It not only enhances the contractile force of the transverse urethral sphincter, but also compensates for the lack of urethral smooth muscle function, so as to improve the urethral pressure. In addition, PFME can provide more support and restraint for the bladder and urethra, thus reducing the over-activity of the detrusor muscle (5, 27).

A number of studies have proved that PFME has a significant improvement effect on postoperative UI, and increasing the intensity of PFME can accelerate the recovery of urinary incontinence (28), but there has been controversy over the starting time of PFME. Our results showed that both perioperative and postoperative PFME helped to reduce the occurrence of UI in the early postoperative period, while there was no significant improvement in 12 months, consistent with Rangganata et al. (29). Compared with postoperative PFME, perioperative PFME did not bring significant benefits on the improvement of patients’ early postoperative UI, which is consistent with the results of Wang et al. and Gislano et al. (6, 12, 28). However, some studies have shown that patients who start to learn and use PFME before surgery can reduce the occurrence of postoperative UI, and the data is statistically significant (29, 30). We reviewed relevant studies and summarized the following two possible reasons: First, patients can fully perceive the contraction of the PFME without pain, to make the PFME more effective after surgery, and thus accelerate the recovery of UI. Secondly, preoperative PFME makes the overall pelvic floor muscle fibers more robust, which makes the damage and influence of RP on PFME relatively small, and thus reduces the occurrence of postoperative UI (7, 9). However, preoperative PFME also has some disadvantages, such as increasing the consumption of patients’ time and energy. It is also easy to cause patients’ anxiety before surgery (31). Whether PFME is necessary before RP needs to be further analyzed by sufficient studies.

There are some limitations in this meta-analysis. First, there is a certain heterogeneity among studies mainly caused by the diversity of PFME schemes in the studies. Other factors included the differences of definition of UI, the intensity and frequency of interventions, and surgical procedures (32, 33). In addition, the included studies could not blind the experimenters and the interveners in this study with PICO, which may affect the results.

## CONCLUSIONS

Application of PFME after RP can significantly reduce the incidence of early postoperative UI, and additional preoperative PFME had no significant improvement on the recovery of UI. Urologists should instruct the patient to perform PFME after removing the catheter, while preoperative PFME is not necessary.
